# Tracing fluid infiltration into oceanic crust up to ultra-high-pressure conditions

**DOI:** 10.1007/s00410-023-02060-6

**Published:** 2023-10-19

**Authors:** Daniela Rubatto, Morgan Williams, Thorsten Andreas Markmann, Jörg Hermann, Pierre Lanari

**Affiliations:** 1 Institute of Geological Sciences, University of Bern, 3012 Bern, Switzerland; 2 Institut des Sciences de la Terre, University of Lausanne, 1015 Lausanne, Switzerland; 3 The Australian National University, Research School of Earth Sciences, Canberra, ACT 2601 Australia; 4 CSIRO, Mineral Resources, Kensington, WA 6151 Australia

**Keywords:** Garnet, Phengite, Oxygen isotopes, SIMS, Subduction, Fluid–rock interaction

## Abstract

Fluid–rock interaction within the altered oceanic crust and across the slab–mantle boundary during subduction facilitates element transfer, but the dynamics of fluid transport and fluid–rock exchange during upward fluid migration are still unclear. A study of metamorphic fluid–rock interaction within a section of subducted oceanic crust was carried out on eclogites and metasediments of the ultra-high-pressure Lago di Cignana Unit (NW Italian Alps). The *P*–*T* modeling of a quartzschist shows that garnet grew during the prograde and sporadically during the retrograde path and that phengite mainly records the peak to retrograde conditions. Microscale geochemical analysis of garnets has revealed a systematic evolution of oxygen isotopic composition with garnet major element zonation, with extreme within-sample core–rim variations in δ^18^O between 18 and 4‰ providing evidence for external fluid influx. Garnet in eclogites and calcschists, as well as garnet cores in quartz-rich lithologies, shows normal compositional zoning, as expected for prograde garnet growth, and a relatively constant oxygen isotopic composition. The outer garnet growth zones within a few metasediments show reverse or discontinuous zoning and progressively lower δ^18^O. Despite major element zoning, the isotopic composition of mica is homogeneous across chemical zoning in one eclogite and one quartzschist, but shows 6‰ variability in another quartzschist. In the underlying Zermatt–Saas serpentinites, antigorite from nine serpentinite samples shows some variation in δ^18^O, with average δ^18^O values for individual samples ranging from 1 to 6‰. These results provide evidence for two main stages of external fluid infiltration: (i) fluids from the dehydration of mafic lithologies entered the sequence at peak conditions around 3 GPa, as indicated by the oxygen composition of intermediate zones of mica and garnet, and (ii) low δ^18^O fluids from serpentinites infiltrated parts of the sedimentary package during exhumation prior to 1.5 GPa, as recorded by the 4‰ garnet outer rims. Samples recording external fluid infiltration are concentrated in the lower part of the sequence, indicating channelized fluid flow, suggesting focused fluid infiltration due to permeability contrasts between metasedimentary and eclogitic lithologies. Channelized fluid flow in the ultra-high-pressure metasediments of Lago di Cignana has not resulted in systematic decarbonation of the metasediments.

**Supplementary Information:**

The online version contains supplementary material available at 10.1007/s00410-023-02060-6.

## Introduction

Subduction zones facilitate long-term, large-scale geochemical cycling through the reincorporation of crustal material into the mantle, coupled with the generation of continental crust by arc magmatism. The distinctive geochemistry of arc magmas is generated by multiple slab components and reflects the contribution of a suite of incompatible and fluid-mobile elements from subducted sediments, altered oceanic crust, and serpentinized lithosphere (Plank and Langmuir 1998; Kodolányi et al. 2011; Bebout 2014; Plank 2014). The magnitude of mass transfer within subduction zones results from the tectonic juxtaposition of chemically diverse lithologies and transient exposure to aqueous fluids (Schmidt and Poli 2003, 2014; Bebout 2014) and eventually melts (Hermann and Rubatto 2014). Metamorphic aqueous fluids are generated in the upper oceanic crust over multiple intervals during prograde subduction (e.g., Peacock 1990; van Keken et al. 2011; Bebout 2014; Schmidt and Poli 2014). The main dehydration reactions during subduction are known (Baxter and Caddick 2013; Ague 2014; Schmidt and Poli 2014) and the rough water budget produced by different rock types can be reasonably modeled (e.g., Hacker 2008; Vho et al. 2020a). However, the dynamics of fluids and the mode of fluid transport and fluid–rock exchange during upward fluid migration are much less clear. In principle, free fluids in a rock mass migrate by pervasive flow around individual mineral grains along interconnected porosity (Ague 2014). Locally, this flow can become channelized along discontinuities and high permeability conduits where higher fluxes are likely to be achieved (Zack and John 2007). Such fluid conduits are more likely to be identified because of the contrast in texture, mineralogy, and composition with the host rock. On the other hand, zones of pervasive flow may remain undetected, despite their high volume and, thus, high capacity to transfer fluids.

Oxygen isotopes can be successfully utilized in investigations of fluid–rock interaction in metamorphic systems due to their predictable temperature-dependent fractionation between mineral and fluid phases (e.g., Wenner and Taylor 1973; Zheng 1993; Valley 2001; Vho et al. 2019). Further, they can be used to assess the extent of interaction with externally derived fluids (Putlitz et al. 2000; Miller et al. 2001; Bebout 2014; Vho et al. 2019, 2020a; Bovay et al. 2021b). In open systems, multiple fluid–rock interaction events typically induce a degree of heterogeneity at the mineral scale, especially at low temperature. In situ analysis can provide information at the scale of mineral zonation, which can then be linked to the temporal evolution of the sample to construct *P*–*T*–time–fluid histories (e.g., Angiboust et al. 2014; Martin et al. 2014; Page et al. 2014; Vho et al. 2020b; Bovay et al. 2021b). Under subduction metamorphic conditions, relatively small shifts in mineral δ^18^O values (of ≈ 1‰) are observed to occur with shifts in temperature in closed systems (e.g., Kohn 1993; Vho et al. 2020a). Greater changes in oxygen isotope composition of mineral zones indicate significant interaction with external fluids which can in turn lead to rock metasomatism (e.g., Russell et al. 2013; Martin et al. 2014; Page et al. 2014; Rubatto and Angiboust 2015; Bovay et al. 2021b). To effectively track fluid–rock interaction, minerals that preserve zoning or different growth stages must be targeted.

We investigated a suite of samples from the ultra-high-pressure locality at Lago di Cignana (NW Italian Alps), which consists of serpentinite, altered oceanic crust and a sediment cover that were metamorphosed at conditions equivalent to ~ 100 km depth. This rock suite represents the deepest-known subducted oceanic sediments and, thus, provides a unique natural laboratory for the study of trace element distribution in high-pressure phases (Spandler and Pirard 2013), as well as fluid-mediated element (van Schrojenstein Lantman et al. 2021) and carbon release (Frezzotti et al. 2011; Cook-Kollars et al. 2014). We analyzed in situ oxygen isotopes in minerals to investigate the record of fluid infiltration events occurring during prograde subduction and early exhumation within the upper oceanic crustal section. Sequential growth zones in garnet and mica were targeted because these two minerals have the potential to record different stages of fluid–rock interaction along the evolution of the host rocks. Garnet commonly grows during prograde metamorphism within both metasedimentary and metabasaltic lithologies, typically at the expense of hydrous minerals and, thus, involving the production of a free fluid phase, making it an effective monitor of dehydration (e.g., Dragovic et al. 2012; Baxter and Caddick 2013). A distinct garnet growth stage can occur during exhumation if temperature increases (e.g., Laurent et al. 2018; Bovay et al. 2021a). Garnet is also mechanically robust and resistant to dissolution. White mica, especially the potassic phengite series, is another ubiquitous mineral in high-pressure rocks derived from sediments and altered basalts. Unlike garnet, mica is a H_2_O-rich mineral that is particularly reactive in presence of fluid resulting in a record of partial re-equilibration stages (e.g., Santamaría-López et al. 2019; Vidal and Parra 2000).

There are several potential sources of metasomatic agents in the upper oceanic crust. In particular, serpentinites contain a large fraction of the bound water in the subducted upper oceanic lithosphere beyond the forearc (Hacker 2008) and are commonly identified sources of high-pressure metamorphic fluids (e.g., Scambelluri et al. 2004; Angiboust et al. 2014). Serpentinites commonly inherit variable δ^18^O compositions from seafloor hydration related to the geological setting. Most abyssal serpentinites have δ^18^O between 3 and 9‰, but they can reach values up to 13‰ (Wenner and Taylor 1973; Skelton and Valley 2000; Mével 2003), and hence potential heterogeneity must be considered. Therefore, in this study, we have also investigated the composition and variation within the serpentinites of the Zermatt–Saas Unit, directly underlying the Lago di Cignana Unit, to assess their potential as a source of metasomatic fluid. This approach makes it possible to distinguish between different stages of external fluid influx and to detect locally variable fluid fluxes related to the structure of the unit.

## Geological setting

The Lago di Cignana Unit is outcropping within the Zermatt–Saas meta-ophiolites, which form the northern part of the Piedmont–Ligurian oceanic units of the Penninic domain in the Western Alps. The Zermatt–Saas zone consists of metaperidotites, metagabbros, metabasalts, and serpentinites (Bearth 1967, 1976; De Giusti et al. 2003), overlain in some places by a thin cover of Jurassic oceanic metasediments (metapelites, marbles and metacherts, Bearth 1967). The Lago di Cignana Unit lies within the upper part of the Lower Unit of the Zermatt–Saas Zone (Forster et al. 2004; Groppo et al. 2009). Peak conditions for the lower unit of the Zermatt–Saas ophiolite are estimated to be about 2.2–2.7 GPa at 550–660 °C (Barnicoat and Fry 1986; Angiboust et al. 2009; Groppo et al. 2009; Rebay et al. 2012; Zanoni et al. 2016).

The Lago di Cignana unit consists of a small fault-bounded block of a coherent segment of former oceanic crust (Reinecke 1991, 1998; Compagnoni and Rolfo 2003) that has been subducted to deeper conditions than the surrounding Zermatt–Saas metaophiolite. It exposes a sequence of metabasalts and metasediments, with minor serpentinites (Reinecke 1991, 1998; van der Klauw et al. 1997) and represents the deepest-known slice of subducted oceanic crust preserved at the surface *(P*–*T* conditions of 590–630 °C and 2.7–3.2 GPa, Reinecke 1998; Groppo et al. 2009), as also supported by the presence of coesite in garnet from quartzites (Reinecke 1991) and microdiamond inclusions in garnet within Mn-nodules (Frezzotti et al. 2011). The relatively intact structure of the unit allows reliable delineation of lithologies and the rapid exhumation has minimized the influence of post-peak re-equilibration (Reinecke 1998).

The Zermatt–Sass ophiolite and overlying sediments have formation ages of 161–165 Ma, based on zircon U–Pb ages of the Mellichen and Allalin metagabbros and the Lago di Cignana metasediments (Rubatto et al. 1998). Most garnet Sm–Nd and Lu–Hf ages indicate prograde to peak metamorphic conditions in the Zermatt–Sass unit between 50 and 43 Ma (Lapen et al. 2003; Skora et al. 2009; Dragovic et al. 2020; Bovay et al. 2021a). This chronology is supported by 44.1 ± 0.7 Ma U–Pb ages of metamorphic zircon in the Lago di Cignana metasediments (Rubatto et al. 1998), as well as Ar–Ar ages (43–45 Ma; Gouzu et al. 2006) and Rb–Sr ages (45–44 Ma; de Meyer et al. 2014) of garnet-hosted white mica inclusions from the broader Zermatt–Saas unit. Matrix phengite records the age of exhumation at 40–36 Ma (Gouzu et al. 2006; de Meyer et al. 2014).

The unit preserves records of fluid cycling at pressures similar to sub-arc conditions. Previous studies have documented prograde devolatilization and decarbonation reactions within metapelites (Bebout et al. 2013; Frezzotti et al. 2011; Cook-Kollars et al. 2014) and fluid-mediated geochemical interactions between serpentinites and sediments (Selverstone and Sharp 2013; Gilio et al. 2019; van Schrojenstein Lantman et al. 2021). Additionally, the isotopic composition of carbonate-bearing metasediments are consistent with infiltration of low δ^18^O fluids, which are likely to be partially involved in decarbonation reactions (Cook-Kollars et al. 2014). Similarly, the variable δ^11^B of mica in the metasediments and eclogites is interpreted to represent fluid infiltration of serpentine-derived fluids at peak conditions and during retrogression (Halama et al. 2020).

## Methods

### EMPA

Major element analyses of garnet were conducted on a Cameca SX-100 electron microprobe with four wavelength-dispersive spectrometers at the Research School of Earth Sciences (RSES), The Australian National University. Analyses were conducted with a 10 nA electron beam, an accelerating voltage of 15 keV, with signals measured on TAP (Na, Mg, Al, Si, P), PET (K, Ca, Ti, Cr, Mn), and LLIF (Ni, Fe, Mn, Cr, Ti) spectrometers, using peak counting times of 10–30 s. Na and K were analyzed first to avoid beam damage effects. A suite of mineral reference materials was used for standardization. Garnet reference material reproducibility for major elements was 0.4–0.8%; reproducibility for the minor to trace components Ti and Cr was 1.6 and 15.6%, respectively. Major element mapping was conducted on a JEOL-8200 electron microprobe at the Institute of Geological Sciences, University of Bern, operated with an accelerating voltage of 15 keV and a 100–200 nA beam focused to a spot of approximately 1 μm diameter. Most major elements were analyzed using wavelength-dispersive spectrometers (WDS; Si, Ca, Fe, Mg, Al), and other elements were analyzed using energy-dispersive spectrometer (EDS; Mn, Ti, La, Ce, Zr, S, K, Na, P). The X-ray maps were acquired using dwell times of 80–150 ms and step sizes of 1–3 μm. Element maps were processed and reduced in XMapTools 3.4.1 (Lanari et al. 2014, 2019).

### LA-ICP-MS

Trace element analyses were performed on a HP Agilent 7700 quadrupole ICP-MS coupled to a 193 nm ArF Excimer laser at 5 Hz repetition rate, 50 mJ fluence, and 47 μm spot size at RSES. Ablation was performed in a He atmosphere using a mixed Ar/H_2_ carrier gas. Torch position and lens tuning were adjusted to maximize signal stability and sensitivity over all masses, while maintaining a low abundance of molecular species (ThO^+^/Th^+^ at or below 0.5%). NIST612 was used as the primary standard (reference values compiled by GeoRem, Dec 2009), with NIST610 and BCR-2G used as secondary reference materials. Standard bracketing was performed with eight sample analyses between each set of standards. Analyses identified as compromised by inclusions (e.g., by monitoring of Zr, P, S, Ti signals for characteristic spikes) were excluded from the dataset. Data below the limit of quantification (3 times the limit of detection) or within 2*σ* of zero were omitted. Data reduction was performed in Iolite 2.3, using the trace element internal standardization routine (Paton et al. 2011).

### SIMS δ^18^O

Garnet oxygen isotope analyses were performed on the SHRIMP II ion microprobe at RSES, using a 15–30 nA Cs^+^ primary beam and a spot size of 25–30 μm. Analyses were performed primarily on grain mounts of garnet separates containing both whole garnets and garnet fragments. Mounts were coated with 50–70 nm of aluminum prior to analysis. Exclusively for sample C13, analyses were carried out on small areas cut from thin sections containing whole garnets. The reference material UWG-2 was used to standardize sample analyses δ^18^O = 5.8‰_SMOW_ (Valley et al. 1995). The uncertainty on the mean value for UWG-2 was ≤ 0.12‰ (± 2SE) for all SHRIMP measurement sessions presented here, and this uncertainty has been propagated for external uncertainty estimates.

Measurement bias associated with variations in target composition (matrix bias) was corrected for using the scheme of Martin et al. (2014), as it was determined on the same instrument shortly before the acquisition of our data. Estimates of matrix bias are expressed relative to the UWG-2 reference material (Valley et al. 1995). The matrix calibration was further tested during the course of this study by analyzing key secondary reference materials during each analytical session. It has been demonstrated that the matrix correction for grossular in the compositional range of the garnet analyzed remains relatively constant between sessions and even between instruments (Martin et al. 2014; Vho et al. 2020c). Some of the samples analyzed contain garnet with particularly high spessartine (which can cause biases of up to 2.3‰; Martin et al. 2014) and this matrix bias was checked by running the spessartine reference materials SPSBH and GRT-1A, with 68% and 93% spessartine, respectively (Martin et al. 2014). The reference material SPSBH was measured in two separate sessions, with weighted average measurements of δ^18^O = 9.56 ± 0.14‰ (2*σ*), 9.96 ± 0.18‰ (2*σ*) (laser fluorination value of 8.13 ± 0.13‰, 2*σ*, Martin et al. 2014). This corresponds to a matrix bias of between + 1.3 and + 1.7‰ (after *X*_grs_ correction), which is comparable to the bias of + 1.6‰ bias reported by Martin et al. (2014). Garnet GRT-1A was measured in one session and yielded a value of 11.3 ± 0.18‰ (2*σ*), corresponding to a matrix bias of 1.1‰, which is within uncertainty of the 1.0‰ bias reported by Martin et al. (2014). The matrix bias associated with *X*_and_ is always below 0.1‰. Matrix bias corrections as described in Martin et al. (2014) were, therefore, applied to the data using EMPA spots located within 20 μm of the SHRIMP spots. The total matrix bias for individual analyses was between 0 and 2.8‰. The uncertainty in the matrix bias correction (± 0.2–0.3‰) is also included in the external uncertainty estimates for individual SHRIMP spots. The total uncertainty of individual garnet δ^18^O matrix-corrected analyses is typically between 0.25 and 0.40‰ (2*σ*). Analyses of standards and reference materials are given in the supplementary tables.

Oxygen isotope analyses of serpentinite were performed on SHRIMP SI at RSES, using a similar sample preparation and analytical routine to that described above for garnet. Analyses were carried out on millimeter-sized chips of rocks mounted in epoxy with the reference material. Analyses were measured in areas free of visible magnetite. The Cerro del Almirez serpentinite Al06-44A was used as the reference material (δ^18^O = 8.3 ± 0.12‰, Scicchitano et al. 2018). The repeatability of the reference material was ± 0.78‰ (2*σ*), and the uncertainty on the mean value of ± 0.13‰ (2SE) was included in the presented data. No bias related to antigorite orientation or compositional variation has been observed (at least within the typical Mg# ranges for ophiolitic serpentinites, Scicchitano et al. 2018), and as the Zermatt–Saas serpentinites are strongly antigorite-dominated, the Cerro del Almirez antigorite provides a well-suited matrix-matched reference material. The average uncertainty of the δ^18^O analyses of individual serpentine samples is 0.20‰, with the maximum uncertainty of individual δ^18^O spots being 0.23‰.

Oxygen isotopes analyses of white mica were performed at the SwissSIMS facility at the University of Lausanne, using a 10 kV primary Cs^+^ beam with a current of ~ 2 nA and a spot size of 10–15 μm. Mounts were checked for topography using a white light interferometer and coated with 35 nm of gold prior to analysis. Samples were mounted as thin section cuts (from which compositional maps had previously been obtained) together with the reference material. Mica UNIL WM-1 (FeO of 1.13 wt% and XMg of 0.57, Luisier et al. 2022) was used as reference material. No compositional matrix effect in O isotope analysis is reported for white mica (Luisier et al. 2022). The repeatability of the reference material in each session was between 0.5 and 0.9‰ (2*σ*).

### Sample description

**Fig. 1:**
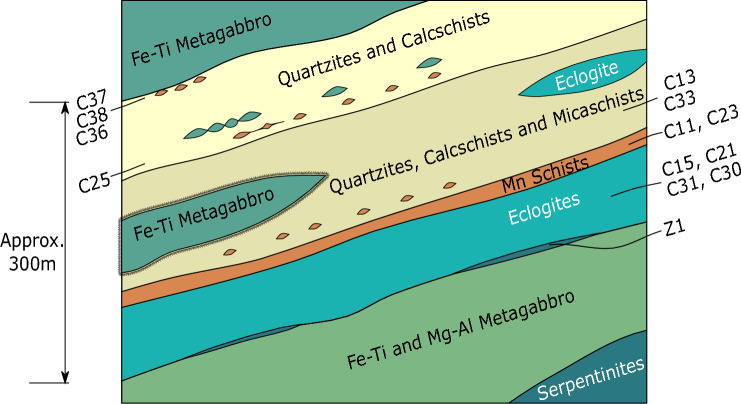
Simplified schematic stratigraphy of the Lago di Cignana Unit, with sample locations. Dimensions in diagram are approximate, but the Lago di Cignana Unit itself is in the order of 300 m thick, from eclogites through to upper quartzites and calcschists (Reinecke 1998; Forster et al. 2004)

Samples of eclogites and metasediments (quartzschists, quartzite, and calcschists) were collected along the southern shore of Lago di Cignana (Fig. 1, Table 1). In addition, a number of serpentinite samples were collected from the underlying Zermatt–Sass unit along a ~ 5 km transect from Perrères to Lago di Cignana. The lithologies present at Lago di Cignana follow a sequence from eclogites through Mn-rich sediments to quartz-rich schists and calcschists (Fig. 1). Mafic lithologies are interbedded with sediments, in places either as small rounded fragments of centimeter size or on a larger scale as meter-sized boudins. Metasedimentary assemblages (Table 1) vary on scales from < 10 cm to meters. While some changes are gradational between lithologies, discrete compositional banding is also observable on the hand-sample scale.

**Table 1:** Main assemblage and location of investigated samples

Lithology (unit*)	Sample	Main assemblage**	Note
Eclogite (LdC)	C15	Grt-Gln-Chl-Rt	
	C21	Omp-Gln-Grt-Ph-Rt	Qz-Ttn vein; Gln more abundant near vein
	C30	Grt-Omp-Ph-Zo/Czo-Gln	
	C31	Grt-Omp-Ph-Gln-Zo/Czo-Qtz-Chl-Rt-Opaques	Minor Qz in Grt pressure shadows and veinlets
Manganiferous Grt-quartzschist (LdC)	C11	Qt-Grt-Ph-Pie-Tur-Braunite/Mag	Banded sample
	C23	Qz-Grt-Ph-Pie-Tur-Braunite/Mag-Chl	Banded sample; possible pseudomorphs after Lws
Grt-quartzschist (LdC)	C37	Qz-Grt-Chl-Phg-Zo/Czo-Tur	Chl in the foliation and in larger aggregates; in contact with garnet nodule
Grt-Czo-Amp-quartzite (LdC)	C13	Qz-Grt-Act-Zo/Czo-Omp-Chl-Ttn-Rt-Ap	Banded sample
Grt-Ph-quartzschist (LdC)	C33	Qz-Grt-Ph-Chl-Cc-Zo/Czo-Ttn-Tur-Ab-Mag-Po-Cpy	Thick Ph bands
	C38	Qz-Grt-Ph-Zo/Czo-Cc-Chl-Tur-Ab-Ox/Hydrox	Large carbonate
Calcschist (LdC)	C25	Qz-Ph-Cc-(Dol)-Chl-Ep-Grt-Tur-Ox./Hydrox.	Dolomite weathered out
	C36	Qz-Cc-Ph-Ep-Chl-Ox-Grt-Tur-Rt	Chl-Qz aggregates replacing Grt porphyroblasts
Serpentinite (ZSZ)	Z1, Z2, Z5, Z6, Z7, Z8, Z14, Z15, Z17	Atg-Mag ± Ti-Clum, Cc	All samples are foliated and contain variable amounts of Mag; Z8 is within a shear zone; Z14 contains bands of Tlc-Tr

For sample locations refer to Fig. 1

*LdC = Lago di Cignana Unit; ZSZ = Zermatt–Saas Zone

**Mineral abbreviations according to Whitney and Evans (2010); Gln indicates blue amphibole identified by optical microscopy

The samples investigated represent the major lithologies of the unit. The stratigraphically deeper samples are the eclogites (C15, C21, C30, C31), followed by the manganiferous quartzschists (C11 and C23) and the overlying quartzitic metasediments (C13 and C33). Sample C25 is a calcschist typical of the middle section in contact with quartzitic metasediments. The uppermost samples were collected within 50 cm of each other from below the metasomatic upper boundary of the unit (calcschist C36, quartzschist C37 and C38). The upper contact of the unit is towards prasinitic material—retrogressed mafic lithologies—with the sedimentary lithologies grading into an undulating/folded metasomatic zone dominated by epidote, chlorite, and white mica.

### Sample petrography

**Fig. 2:**
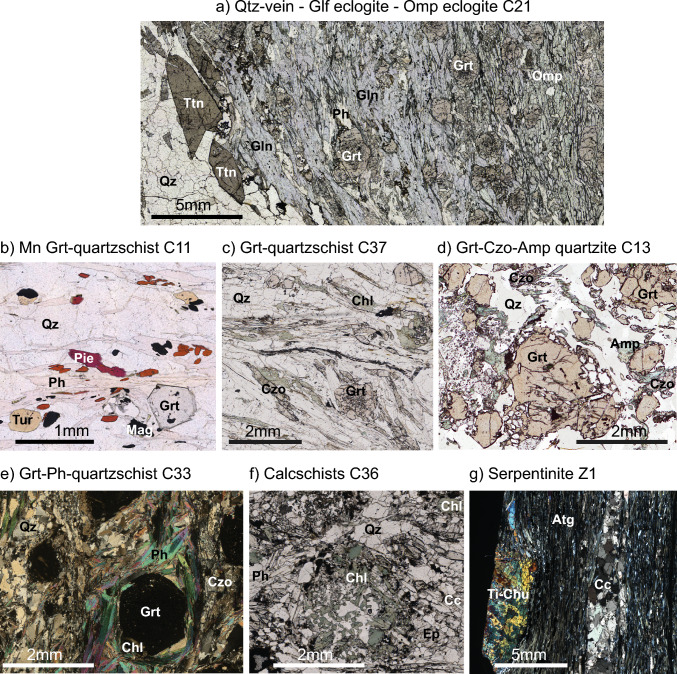
Photomicrographs of thin sections showing major petrographic features. **a** Compositional banding in eclogite C21, from omphacite-rich (right) to glaucophane-rich (center) and to a titanite-bearing quartz vein (left). **b** Manganiferous Grt-quartzschist C11 with small idiomorphic garnets in a quartz-dominated matrix. Garnet, piemontite, braunite, and tourmaline are concentrated within bands delineated by Mn-bearing phengite to form a rough planar fabric. **c** Dominant assemblage within Grt-quartzschist C37 with small- to medium-sized garnets in a quartz-dominant matrix, a fabric defined by phengite with zoiste/clinozoisite and aggregates of chlorite. **d** Grt-Czo-Amp quartzite C13 with coarse garnets in a quartz-rich matrix. Intergrowths of amphibole–clinozoisite–albite are typically found adjacent to garnet. **e** Phengite wrapping around inclusion-bearing garnets in Grt-Ph-quartzschist. **f** Common textures observed in calcschists, including a garnet partially replaced by chlorite. **g** Ti-clinohumite aggregate and carbonate vein within the foliated antigorite serpentinite Z1, found at the base of the Lago di Cignana Unit. Mineral abbreviations according to Whitney and Evans (2010)

*Mafic eclogites* (C15, C21, C30, and C31) consist of omphacite, glaucophane (rimming omphacite or forming isolated crystals in omphacite-dominated matrix), garnet, clinozoisite/zoisite, phengite, paragonite, rutile and minor retrograde blue–green amphibole, chlorite, and oxides (Table 1). The relative proportions of omphacite and glaucophane (both stable at the UHP peak, Groppo et al. 2009) can vary considerably and the samples show banding. In sample C31, white mica and omphacite define a foliation wrapping around garnet. Garnet crystals in all samples are typically large (> 300–500 μm) and have inclusion-rich cores; inclusions of phengite and rutile are the most common, with rutile generally concentrated in garnet rims. Quartz is rarely present as a minor phase either in garnet pressure shadows or in veins (C21, Fig. 2a).

*Manganiferous garnet quartzschists* (Mn-Grt-quartzschists, C11 and C23) exhibit a matrix of quartz, phengite, piemontite (Mn-rich epidote) and garnet (Fig. 2b); accessory minerals are tourmaline, locally abundant braunite-magnetite and minor apatite (Table 1). The Mn-quartzschists are typically devoid of carbonates. Aggregates of fine-grained phengite with minor braunite-magnetite, piemontite and garnet are found within the fabric defined by quartz and larger phengite; the aggregates may represent pseudomorphs after lawsonite. Garnets are typically small (< 500 µm) and idiomorphic. Garnet has inclusions of quartz, Fe ± Mn oxides, and zircon throughout the zones, with phengite and rutile commonly present in the outer zones.

One sample of garnet *quartzschist* (Grt-*quartzschist,* C37) was collected adjacent to large metamorphosed manganese nodules (cm–dm in diameter, Fig. 2c). It contains small, zoned garnets (< 100 μm to 500 μm) within a quartz-dominated matrix; a moderately folded fabric is defined by phengite, zoiste/clinozoisite, and chlorite. Chlorite forms larger lenticular aggregates. Minor carbonate and albite are also present. Aggregates of phengite, garnet, minor chlorite and opaque oxides follow the phengite-defined fabric. Accessory phases include dravitic tourmaline (Williams 2019), sulfides, and zircon. Nodules contain inclusion-rich, reddish garnet within a network of quartz, with zones dominated by quartz containing larger garnets, usually with few inclusions and minor phengite; these nodules are similar to those studied by Frezzotti et al. (2011) and van Schrojenstein Latman et al. (2021). The boundary between the quartzschist and the garnet nodule is marked by phengite, amphibole ± talc.

The *garnet clinozoisite amphibole quartzite* (Grt-Czo-Amp-quartzite C13) is banded on the cm-scale, with domains rich in quartz and mafic minerals alternating at the centimeter scale. This feature is interpreted as an interlayering of quartz-rich sediments with mafic detritus. The sample contains millimeter-sized garnets with concentric zones and domains of pink color in plain polarized light, related to Mn-rich garnet, embedded in a quartz-rich matrix (Fig. 2d). A fine-grained intergrowth of bluish-green amphibole, zoisite/clinozoisite and albite is interpreted as a pseudomorph after omphacite. Needles of unoriented blue–green amphibole are common. Allanite occurs as an inclusion in garnet rims and only rarely in the matrix, where it is overgrown by clinozoisite. Clinozoisite occurs as small laths in the matrix or as irregular grains associated with amphibole, quartz, and albite. The latter are confined to restricted domains that are interpreted as lawsonite pseudomorphs. Rutile is present as inclusions in garnet, while in the matrix it is replaced by titanite. Minor chlorite replaces garnet (Fig. 2d). A crystallization diagram of minerals with different metamorphic stages is given in Supplementary Fig. 1S.

Two *garnet phengite quartzschists* (Grt-Ph-quartzschist, C33 and C38) were sampled from either end of the stratigraphic section, both consisting of quartz, phengite, paragonite, garnet, chlorite, zoisite/clinozoisite, carbonate (more abundant in C38, rare in C33), minor albite, amphibole, titanite and rare rutile (Table 1). A foliation defined by phengite and quartz (Fig. 2e) wraps around the idiomorphic garnet porphyroblasts. Sample C33 has a greater phengite modal abundance (Fig. 2e), has rhombic aggregates of phengite (mica fish) aligned with the fabric and smaller phengite grains within the foliation. Garnet grains are generally dominated by large cores (300–500 μm across), and rims and intermediate zones (10–300 µm) show variable retrogression and alteration to chlorite, resulting in the formation of chlorite-rimmed garnet in some samples and locally the formation of atoll garnet. Outer zones of garnet contain inclusions of quartz, rutile, dolomite, zircon and apatite, with micas observed only in the outer rims. In sample C38 there are fine-grained intergrowths of white mica, albite and quartz in confined domains with the foliation wrapping around, probably representing lawsonite pseudomorphs. This texture is absent in the more micaceous sample C33. Sample C38 contains millimeter-sized, sometimes idiomorphic, carbonate blasts that are coated with Fe-oxides along cracks and borders. The Fe-bearing carbonates in C33 are smaller, more altered, and display an irregular shape. A crystallization diagram of minerals with different metamorphic stages for sample C33 is given in Supplementary Fig. 1S.

*Calcschist* samples (C25 and C36) are dominated by a matrix of quartz and calcite interspersed with phengite and epidote/clinozoisite (together defining a planar fabric). The matrix assemblage varies from quartz–phengite dominated (with a stronger foliation) to carbonate ± phengite dominated. The amount of garnet in these samples is minor, mostly due to retrogression. In fact, sample C36 contains aggregates of quartz and chlorite that probably formed after previous garnet porphyroblasts (Fig. 2f). Accessory minerals include Fe-oxyhydroxides after former dolomite, and rare dravite and rutile.

Samples taken from the underlying Zermatt–Saas unit are planar-foliated *antigorite serpentinites* containing variable amounts of magnetite. The serpentinites show variations in grain size, fabric and also magnetite abundance. The uppermost serpentinite adjacent to the Lago di Cignana Unit (Z1) shows large grains of elongated antigorite, rare nodules of titanian clinohumite (~ 4 wt% TiO_2_), and localized calcite veins forming a planar fabric (Fig. 2g). Other Zermatt–Saas serpentinites exhibit poorly oriented antigorite interspersed with up to ~ 5 vol% magnetite. One sample has locally concentrated magnetite intergrown with serpentine (Z15) and another is partially altered to a talc–tremolite assemblage (Z14).

### Garnet composition

**Fig. 3:**
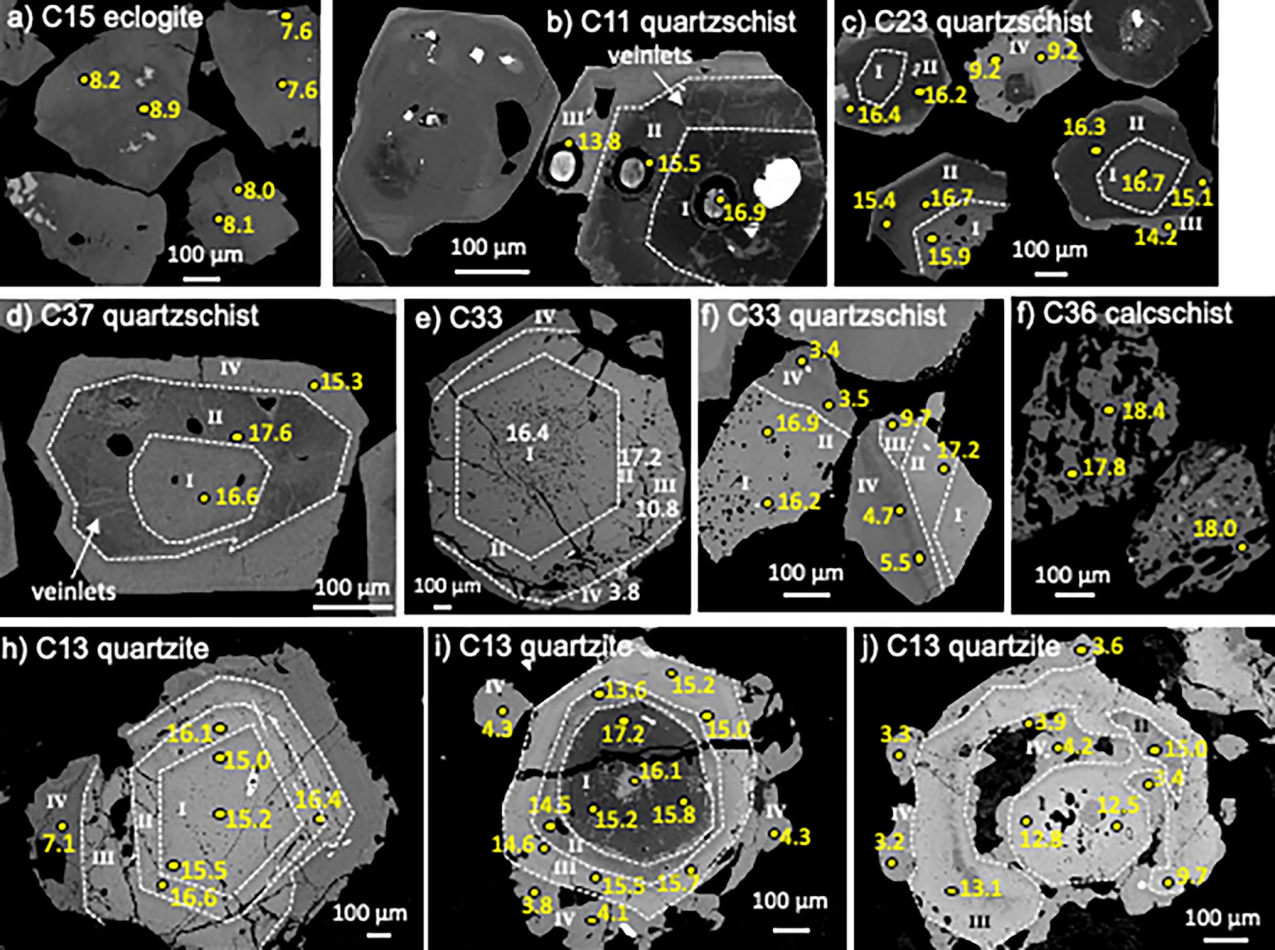
BSE images of typical garnet zoning. Garnet images **a**–**j** show positions of oxygen isotope analyses with δ^18^O (‰). Garnet zones are marked with roman numbers I–IV and their boundaries are marked with white dashed lines. In **e** the average zone δ^18^O is given as this grain (the same as shown in Supplementary Fig. 3S) was not analyzed, but is the best representation of the garnet zones

A range of garnet compositions is observed between and within individual lithologies at Lago di Cignana (Table 2, Supplementary Fig. 2S and 3S, Supplementary Table 1S; Williams 2019). Many of the lithologies contain garnet with characteristic zoning patterns (e.g., Figure 3). Two main types of garnet can be distinguished: (i) garnet from mafic eclogites and calcschists has a relatively smooth unidirectional zoning from core to rim; and (ii) garnet from quartz-rich metasedimentary samples shows a strong compositional contrast between individual garnet growth zones that truncate each other (see details below). In metasediments with compositional layering, spatial modulation of garnet grain density and size (from thin section to outcrop scale) is present. For this reason, garnets from the Grt-Czo-Amp-quartzite have been divided into several groups to reflect different local garnet growth systematics (Table 2).

**Table 2:** Mean normative garnet composition and oxygen isotope compositions of samples, and where applicable, individual growth zones

Sample	Garnet zone	Prp	Alm	Grs	Sps	And	No. of analyses	δ^18^O‰ range	Mean δ^18^O‰	± 2 SD
Eclogites										
C15		17.1	59.9	19.7	1.9	1.3	7	7.6–8.9	8.1	0.9
C21		13.5	60.8	22.1	2.2	1.4	12	7.1–8.2	7.6	0.7
C30		17.5	61.1	18.6	1.6	1.2	9	7.8–8.7	8.2	0.6
C31		18.8	63.6	15.8	0.7	1.1	10	7.8–9.4	8.5	1.1
Mn-Grt-quartzschists										
C11	I	29		11	60	0.5	5	15.0–16.9	15.8	1.4
	II	22	< 0.1–3.2	13	64	0.6	7	13.8–16.3	15.2	1.9
	III	14	< 0.1–27	15	66	0.9	6	13.0–15.8	14.3	2.1
	IV	6	1	14	79	0.3	1		14.2	
C23	I	27.5		10	62	0.5	5	15.8–16.7	16.3	0.8
	II	29		10	60	0.5	9	15.4–16.7	15.9	0.9
	III	10		11	78	0.4	2	14.2–15.3	14.9	1.6
	IV	1.2		11	86	1.3	3	9.2–11.5	9.9	2.2
Grt-quartzschist										
C37	I	17	13	11	60	0.7	9	16.4–17.3	16.8	0.6
	II	19	30	13	58	0.9	7	17.0–17.6	17.4	0.5
	III	15	38	13	45	0.9	3	16.1–16.2	16.2	0.2
	IV	10	48	16	24	1.3	2	15.3–16.3	15.8	1.3
Grt-Czo-Amp-quartzite										
C13-G1 + 13	II	8	53	27	12		3	9.7–13.9	11.7	4.2
	III	9	53	33	3		5	9.2–9.7	9.5	0.4
	IV	9	51	35	4		13	4.7–2.5	3.8	1.2
C13-G4 + 11	I	3	49	25	23		7	12.6–15.5	14.8	2.1
	II	10	66	19	6		7	16.1–17.5	16.5	0.9
	III	8	51	36	4		7	8.9–4.2	7.1	2.9
C13-G2	I	33		10	54		3	15.2–17.2	16.1	2.1
	II	15	2	18	63		2	13.6—14.5	14.0	1.2
	IIb	7	51	23	18		4	14.5–15.6	15.2	1.0
	IV	8	51	37	3		4	3.8–4.3	4.1	0.6
C13-G5	I	31	1	10	57		2	13.3–13.5	13.4	0.2
	II	13	3	17	67		3	13.4–15.3	14.1	2.1
	III	10	58	29	3		10	6.9–9.0	7.6	1.7
	IV	8	53	32	7		3	3.8–4.1	3.9	0.3
C13-G6	I	10	53	22	14		2	12.0–12.2	12.1	0.4
	II	13	33	21	33		3	13.5–14.0	13.4	0.5
	III	9	57	30	3		7	7.1–8.5	7.5	1.0
	IV	10	50	34	6		3	3.5–4.2	3.9	0.7
Grt-Phe-quartzschist										
C33	I	4.7	57.3	22.2	14.9	0.9	16	15.5–18.4	16.4	1.7
	II	7.1	65.9	17.3	8.8	1.0	9	16.5–17.9	17.2	0.9
	III	10.0	61.7	22.0	5.4	1.1	18	7.1–13.5	10.8	4.2
	IV	12.5	53.3	22.4	10.3	1.4	10	2.2.–5.5	3.8	1.9
C38		6.0	64.9	20.2	7.5	1.3	10	16.2–18.3	17.4	1.7
Calcschists										
C25		4.5	41.5	24.8	28.1	1.2	16	15.5–18.3	17.2	1.4
C36		4.3	42.2	24.5	27.5	1.8	11	17.9–18.7	18.2	0.6

Garnet normative compositions have units of mol%. Elemental abundances below detection limit are not shown

Refer to the Supplementary Table for more details on the variability of garnet major element composition for each zone

Almandine–grossular garnet is typical of the eclogites and Grt-Ph-quartzschists, with variable pyrope content up to 20%. On the other hand, the Mn-Grt-quartzschists and the Grt-quartzschist contain spessartine-rich garnet (*X*_sps_ 24–86). Garnet in the Grt-Czo-Amp-quartzite is extremely zoned and generally has a high spessartine content. The calcschists contain grossular–spessartine garnet. Andradite is a minor component in all samples, which may increase in abundance within the outer zones (always < 2%, as estimated from stoichiometry).

Normal compositional zoning (i.e., a bell-shaped Mn profile; Supplementary Fig. 3S; Hollister 1966) is observed within inner metamorphic garnet zones of all samples. Inverted compositional zonation (where *X*_sps_ increases outwards) is found in several samples, typically comprising the outer individual growth zones. The transition between normal and inverted compositional zonation is sharp and typically marked by a significant jump in composition. In addition to the general description above, the characteristics of each rock type are outlined below.

*Eclogitic* garnets from samples C15, C21, and C31 are dominated by Alm (consistently > 55%) and the outward growth zoning of garnets is mainly towards more Prp-rich compositions at the expense of Grs. Garnets from samples C31 and C15 exhibit visible veinlets or “healed fractures” similar to that reported for other eclogitic garnets (Angiboust et al. 2011; Rubatto et al. 2020). Garnet in sample C30 has a thin rim enriched in Mn (Supplementary Fig. 3S).

Garnets from *Mn-Grt quartzschists* exhibit four distinct zones (I–IV from core to rim) that can be defined based on textures and composition. This zoning is comparable to what was reported by Reinecke (1998) (Table 2, Fig. 3). In sample C11, four main garnet growth zones are observed, each becoming increasingly Prp-poor (29–6%) and Sps-rich outwards (60–80%, Table 2). The increase in Mn from core to rim is related to the presence of braunite in the sample, which may supply Mn during prograde metamorphism. In sample C23 the garnet cores are among the most spessartine-rich of all samples. The pyrope normative component is high in the core (27–29%) and is low in the rim (10–1%), which is spessartine-rich (78–86%). Garnet from the *Grt-quartzschist* (C37) shows four main zones, which are relatively similar to the Mn-Grt quartzschists (Table 2, Fig. 3). Typical is the decrease in spessartine from core to rim (60–24%) and a fine network of linear (orthogonal to radial) veinlets in the garnet cores, which are enriched in Alm–Grs components (Fig. 3d).

Garnet from the *Grt-Czo-Amp quartzite* C13 is particularly complex and dramatically zoned, and the zonation does not necessarily correlate across grains (Fig. 3). Examples of the garnet compositional variation across grains are given in Table 2, and further details are given in Supplementary Table 2S. Notably, the composition of the outer garnet zone (zone IV, Table 2) converges in all grains.

*Grt-Ph*-quartz*schist* C33 shows four garnet growth zones (Table 2, Fig. 3) with an increasing pyrope component outwards (5–13%). Idiomorphic garnet cores show normal zoning towards more Alm-rich compositions, terminating in sharp boundaries with zone II. Zone III contains the lowest spessartine content and has an undulated diffuse boundary. The outermost rim (zone IV) is characterized by an enrichment in *X*_sps_. *Calcschist* garnets (C25, C36) are ubiquitously inclusion rich (quartz and calcite), resulting in a spongy texture, and have Alm–Sps–Grs compositions with no distinct growth zones.

### Garnet oxygen isotopes

**Fig. 4:**
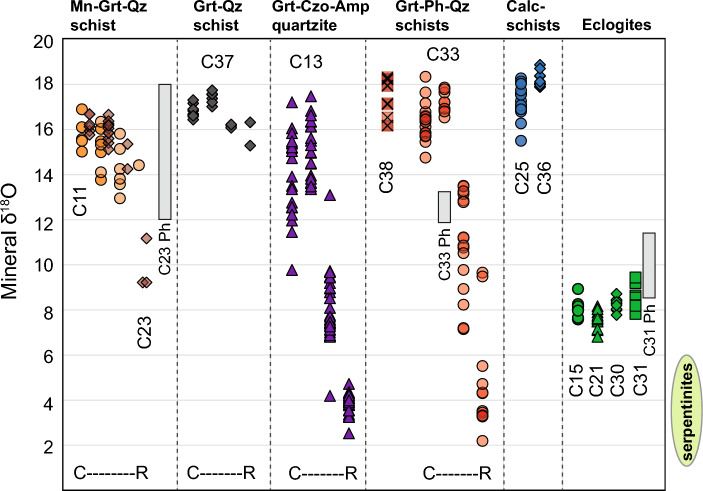
Garnet oxygen isotope zoning by lithological group. Symbols represent individual garnet analyses of different zones and are arranged from core to rim (C–R) in samples where zoning is present. The vertical grey boxes represent the range of δ^18^O of phengite (Ph) in samples C23, C33, and C31. The composition range of antigorite in serpentinites is represented by the green ellipse to the right of the diagram

In situ garnet oxygen isotope compositions were measured for metasediments and eclogites. Between 7 and 95 analyses were obtained for each sample, depending on the degree of heterogeneity and the availability of suitable spot locations (Fig. 3). Overall, the oxygen isotope composition is highly variable between and within samples, with mean δ^18^O values in individual growth zones ranging from 18.2‰ in garnet cores down to 3.8‰ in some garnet rims (Fig. 4, Table 2, Supplementary Table 2S).

Garnet cores in the metasediments have average oxygen isotope compositions ranging from 13 to 18‰, with outward zonation typically showing either minor δ^18^O increases (of maximum 1.5‰, e.g. C33 and some grains in C13) or consistent decreases in zone-average δ^18^O with subsequent garnet growth. The observed isotopic zonation of metasedimentary garnet is generally step-wise, and not all successive compositional garnet zones are associated with oxygen isotope offsets (Fig. 4). Notably, the lowest δ^18^O values are always observed in the outer garnet rims (e.g., zone IV in C13 and C33). Coupled variation between garnet major element chemistry and δ^18^O values is observed between garnet growth zones in a number of samples. The Grt-Czo-Amp-quartzite and Grt-Ph-quartzschist C33 are dramatically zoned in δ^18^O covering the entire spectrum from ~ 18 to 4‰, also reflecting the strong chemical zoning. The Mn-Grt-quartzschists have significant core-rim garnet zoning between δ^18^O of 16 and 14‰ (sample C11) and between 16 and 10‰ (sample C23), respectively. Grt-quartzschist C37 has a restricted garnet isotopic composition (δ^18^O of 17 –16‰) despite having a pronounced chemical zoning in spessartine and pyrope components (Table 2). The calcschist samples are the sediments with the most homogeneous garnet in terms of chemical and oxygen composition with an average a 17–18‰ in both samples.

Concurrent with the limited chemical zoning, the oxygen isotope compositions of garnet from eclogites show consistent compositions of 7.6 to 8.5‰, with relatively little variation within individual garnets (maximum ranges of 1.6‰). Notably, these values are significantly higher than that of garnet from a few eclogites in the Zermatt–Saas unit that have δ^18^O from 4.3 to 5.4‰ (Cartwright and Barnicoat 1999).

### Mica chemical composition and oxygen isotopes

**Fig. 5:**
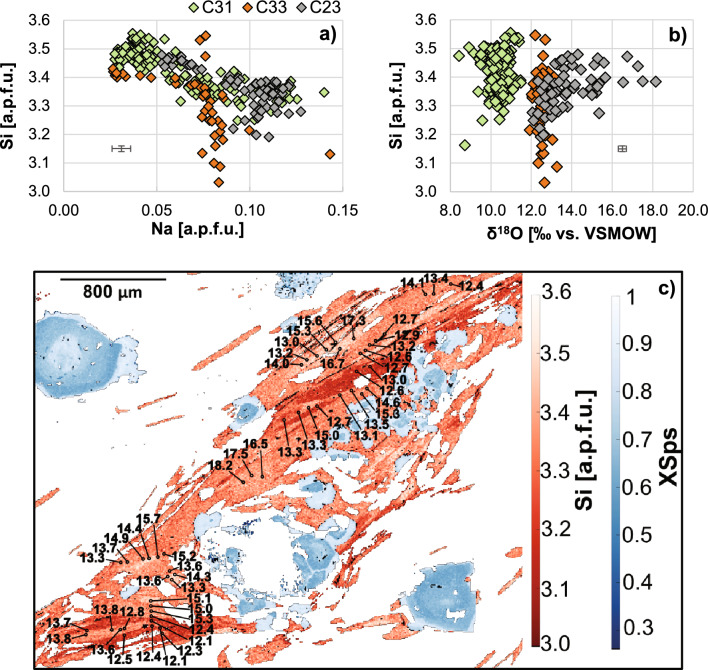
Compilation of **a** mica composition in Si and Na content and **b** oxygen isotope composition vs. Si content for eclogite C31 and quartzschists C33 and C23. Values of major element composition are extracted from X-ray maps using XMapTools (Lanari et al. 2014). Error bars represent the average internal error (2SE) for the oxygen isotope composition from SIMS measurements and the respective major element composition extracted via XMapTools. **c** Representative in situ oxygen isotope results for sample C23 stacked on the Si [a.p.f.u.] X-ray map of phengite (red color bar) and *X*_sps_ map of garnet (blue color bar) processed with XMapTools

Phengite shows a wide range of compositions ranging along the muscovite–celadonite join and complex zoning in the three samples investigated by EPMA mapping (C31 eclogite, C23 and C33 quartzschists; Fig. 5, Table 3 and Supplementary Table 3S). The compositional variability is related to three cation exchanges, the paragonite–muscovite, the Tschermak and Fe–Mg exchange. The chemical variability of phengite can be described using Si- and Na-cations (in atoms per formula unit) and XMg (%) (Table 3, Fig. 5). At least two phengite groups can be distinguished in all three samples. Group A is identified as a high-Si (Si = 3.45–3.53 apfu) and low-Na phengite that is present as large flakes or as cores within the mica defining the foliation. Smaller mica flakes that define the main foliation have lower Si contents of Si = 3.3–3.4 (group B). High Si content in mica generally correlates with higher XMg and lower Na content. In the quartzschist samples, a group C phengite with the lowest Si content represents a minor population that replaces group B phengite at the rim.

**Table 3:** Major element composition and oxygen isotope composition of phengite

Sample	Zone	Si (apfu)	Al (apfu)	Na (apfu)	K (apfu)	XMg	Xmusc	Xcel	FeO	N δ^18^O analyses	δ^18^O‰ range	Median δ^18^O‰	± 2 SD
C33 Grt-Phe-quartzschist													
	Total	3.05–3.50	1.88–3.45	0.02–0.14	0.67–0.97	0.20–0.81	0.41–0.85	0.06–0.36	1.3–4.5	44	11.9–13.3	12.5	0.6
	A	3.37–3.50		0.01–0.05		0.65–0.80					12.2–13.1	12.6	0.5
	B	3.23–3.48		0.05–0.10		0.60–0.81					12.1–12.6	12.2	0.5
	C	3.05–3.22		0.07–0.13		0.3–0.7					11.9–13.3	12.6	0.8
C23 manganiferous Grt-quartzschist													
	Total	3.10–3.55	1.6–2.4	0.03–0.15	0.72–1	0.40–0.85	0.37–0.65	0.14–0.43	2.14–3.95	65	12.1–18.1	13.5	2.8
	A	3.40–3.57		0.04–0.08		0.7–0.8					12.1–18.1	14	2.9
	B	3.23–3.47		0.08–0.15		0.48–0.74					13.2–17.3	13.1	1.7
	C	3.10–3.28		0.05–0.12		0.3–0.6					12.4–12.6	12.5	0.2
C31 eclogite													
	Total	3.2–3.6	1.85–2.40	0.02–0.14	0.83–1	0.3–0.9	0.31–0.55	0.12–0.44	0.16–3.9	184	8.4–11.5	10.4	1.1
	A	3.4–3.6		0.02–0.06		0.72–0.90					8.4–11.4	10.4	1.0
	B	3.20–3.42		0.06–0.14		0.25–0.75					8.7–11.5	10.4	1.1

In situ oxygen isotope compositions of phengite in the three samples show a wide range of δ^18^O values between 8 and 18‰, with a distinction between eclogite and quartzschists (Table 3). Eclogite C31 has the lowest δ^18^O values between 8.4 and 11.5‰ (average 10.4 ± 1.1‰, 2SD) and there is no difference in δ^18^O between high- and low-Si phengite. Phengite in quartzschist C33 is the most homogeneous and has higher δ^18^O values ranging from 11.9 to 13.3‰ (average 12.5 ± 0.6‰). Quartzschists C23 contains mica with the greatest within-sample variability and highest δ^18^O, ranging from 12 to 18‰. In this sample, the highest δ^18^O was measured in mica with higher Si and lower Na contents (Fig. 5).

### Serpentine oxygen isotopes

In situ oxygen isotope analysis of serpentine was performed on nine samples along the 5 km transect from the border of the Lago di Cignana unit (Z1) inside the Zermatt–Saas unit (Supplement Table 4S). Antigorite within each sample presents variations in δ^18^O of 0.3–1.7‰, with the exception of sample Z6 that has a stronger variability over 3.8‰. Average δ^18^O values for individual samples vary from 1.1 to 6.1‰. The samples closer to Lago di Cignana unit have higher δ^18^O values of 3–6‰ that jump to of 1–2‰ after the shear zone. The sample closest to the Lago di Cignana unit exhibits the highest δ^18^O composition, whereas the lowest value is measured in sample Z14 that contains talc–tremolite bands.

### Phase equilibrium modelling of micaschist

Quartzschist sample C33 contains garnet with a large δ^18^O shift from 18 to 4‰ and minerals that record several stages of the *P*–*T* history. Therefore, this sample was selected for phase equilibrium modelling in an attempt to determine the conditions of garnet and phengite stability and to compare them with major fluid producing reactions.

**Fig. 6:**
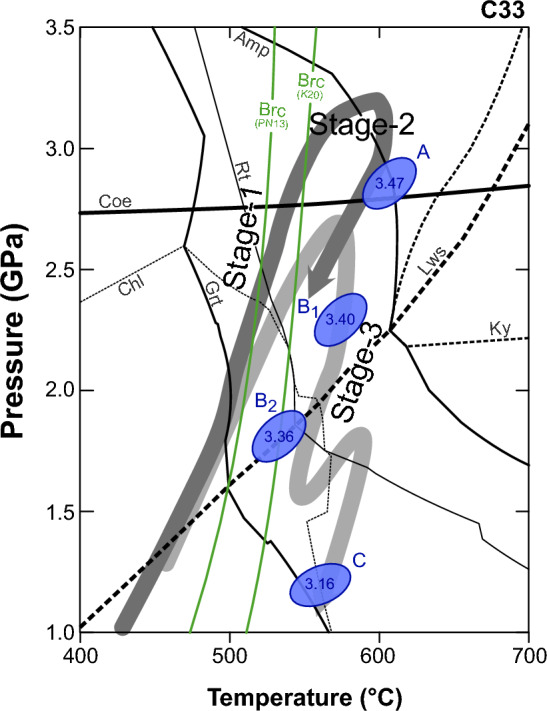
Thermodynamic modeling results for sample C33 showing the stability field of chlorite (Chl), garnet (Grt), rutile, (Rt), amphibole (Amp), lawsonite (Lws), kyanite (Ky), and coesite (Coe). Blue ellipses indicate the formation of phengite of different compositions (Si apfu 3.47 to 3.16; see text). The green lines indicate the breakdown of brucite (Brc) according to the model of Padron-Navarta et al. (2013) and Kempf et al. (2020). The thick arrowed lines are the P–T path of Groppo et al. (2009) for Lago di Cignana (dark gray) and of Bovay et al. (2021a) for the Theodul Glacier Unit (light gray)

A mineral assemblage diagram was calculated for the bulk-rock composition of C33 (Supplementary Table 5S) using the Gibbs energy minimization program Theriak–Domino (de Capitani and Brown 1987; de Capitani and Petrakakis 2010). Calculations were performed in the chemical system Na_2_O–CaO–K_2_O–FeO–MgO–Al_2_O_3_–SiO_2_–TiO_2_–H_2_O using the thermodynamic database tcdb55 (modified after Holland and Powell 1998). The main curves delimiting the stability fields of garnet, chlorite, lawsonite, kyanite, amphibole, coesite, and rutile are shown in a simplified *P*–*T* diagram (Fig. 6). Mineral abbreviations are from Warr (2021). As the model was calculated for a Mn-absent system, the garnet-in and chlorite-out curves indicate the highest *P*–*T* conditions at which garnet should have formed in this rock.

Several attempts were made to determine the formation conditions of garnet using the program GrtMod (Lanari et al. 2017) and the database tcdb55 in the chemical system (MnO–)Na_2_O–CaO–K_2_O–FeO–Fe_2_O_3_–MgO–Al_2_O_3_–SiO_2_–TiO_2_–H_2_O. However, it was not possible to model the observed garnet composition for the core and mantle within the *P*–*T* range (residual values *C*_0_ > 0.20; see Lanari et al. 2017). This lack of agreement between model and observations indicates either that the garnet grew out of equilibrium (e.g., Spear and Wolfe 2019), or could be the result of a mismatch between the present-day bulk-rock composition—used for modelling—and the reactive bulk composition that controlled the garnet composition during growth. Despite this issue, garnet compositional zoning in sample C33 indicates prograde to pressure-peak growth for core and mantle with a possible rim crystallization during exhumation, as previously proposed by Reinecke (1998) for a Lago di Cignana sample and documented in the metasediments of the nearby Theodul Glacier Unit by Bovay et al. (2021a).

The *P*–*T* conditions of formation for four groups of phengite (with Si ranging from 3.47 to 3.16 apfu, Fig. 6 and Supplementary Fig. 4S) were determined from the intersection of Si-isopleths and the *P*–*T* lines representing the phengite-quartz-water equilibrium conditions calculated using the method of Dubacq et al. (2010) and the program ChlMicaEqui (Lanari 2012). This combined approach was used to reduce the potential uncertainty due to open-system behavior. Isopleth thermobarometry of Si-in-phengite is known to be largely unaffected by changes in the reactive bulk composition caused by garnet fractionation (Airaghi et al. 2017) and the multi-equilibrium approach does not require the knowledge of the reactive bulk composition. The results indicate that phengite partially re-equilibrated during exhumation under different pressure conditions from 2.9 to 1.2 GPa at temperatures between 500 and 600 °C (Fig. 6).

## Discussion

Zoned metasedimentary garnet within the Lago di Cignana Unit preserves a wealth of geochemical information that can be related to metamorphic processes. The discussion focuses on the extreme zonation of garnet δ^18^O values and the presence of garnet rims with δ^18^O values approximately 14‰ *lower* than the garnet cores. Notably, this is in stark contrast to the metamorphic trend expected for closed-system metapelites (shift of ≈ + 1‰; Kohn 2014; Vho et al. 2020a), and requires infiltration of externally derived fluids with a high time-integrated fluid flux. Mica, which preserves more moderate zoning in oxygen isotopes, is used for comparison with garnet to assess the equilibration of the matrix. The discussion below highlights some of the details and major implications of the garnet and phengite geochemical records, including potential fluid sources and the context of metasomatism relative to the well-constrained *P*–*T*–*t* history at Lago di Cignana.

### Garnet growth record

The garnet populations analyzed are from heterogeneous lithologies that are likely to record different stages, timing and degrees of open-system behavior. During normal prograde growth of garnet, thermodynamic models and natural observations show that *X*_prp_ increases with temperature, *X*_sps_ decreases with increasing temperature and pressure and the amount of garnet, producing a typical ‘bell-shaped’ profile (Hollister 1966; Spear 2003; Kohn 2014; Lanari and Engi 2017; Konrad-Schmolke et al. 2008). Overall, prograde garnet should become more magnesian with increasing temperature, even if there is significant garnet fractionation (Lanari and Engi 2017). Sharp step-wise changes in garnet chemistry, may be related to major changes in the coexisting mineral and fluid phases during the prograde history, including garnet resorption caused by external fluids, lawsonite breakdown during exhumation or breakdown of minor and accessory minerals (e.g., Reinecke 1998; Konrad-Schmolke et al. 2008; Giuntoli et al. 2018). The range of garnet zonation patterns observed at Lago di Cignana reflects multiple garnet growth reactions and varying degrees of open-system behavior, as discussed below.

Normal (i.e., bell-shaped Mn profile; Hollister 1966) garnet growth zonation is well preserved at Lago di Cignana, particularly in eclogitic garnet and in cores of metasedimentary garnets (Supplementary Fig. 3S). The major element zoning is sharp and, thus, changes in growth chemistry through diffusion-induced re-equilibration are minimal in these samples, due to the relatively low temperature reached during Alpine metamorphism (550–600 °C). Confirming the observations of Reinecke (1998), only early garnet cores may exhibit some degree of diffusional relaxation; all outer garnet zones described here (Fig. 3) show either sharp boundaries or undulating ill-defined boundaries with outer inversely zoned garnet that are attributed to resorption. The retention of sharp compositional boundaries for garnet major element profiles and significant isotopic heterogeneity suggests that garnet did not remain at temperatures greater than 600 °C for extended periods of time (i.e., < 10 My; Crank 1975; Caddick et al. 2010), although it may have had protracted histories at lower temperatures, as suggested by some geochronological studies (Skora et al. 2009).

The garnet-in reaction in the investigated samples probably involved the consumption of chlorite (Spear 2003). The isochemical phase diagram calculated for sample C33 suggests garnet formation on the prograde path above 450 °C and 1.5 GPa (Fig. 6), in line with results obtained in similar rock types in the Franciscan Complex, Alps and in the Cyclades (e.g. Page et al. 2014; Laurent et al. 2018; Bovay et al. 2021a, b). However, the stability of the garnet-chlorite assemblage is extended to lower *T* in Ca- and Mn-rich bulk compositions (Spear 2003), and thus garnet in the generally Mn-rich Lago di Cignana metasediments could have started to grow at even lower *T*. Further garnet growth in metasediments at higher temperatures typically involves phases such as amphibole, mica, epidote, lawsonite and aluminosilicates (e.g., Page et al. 2007; Lanari and Engi 2017; Loury et al. 2018). Garnet with normal growth zoning is present in the eclogite samples and in the core of the metasedimentary garnet, which are relatively homogeneous in oxygen isotopic composition. These garnet types are, therefore, interpreted as the product of prograde growth in relatively closed system that released fluids. In the manganiferous and phengite-rich quartzschists, the growth of internal garnet zones was already recognized by Reinecke (1998) as prograde to ultra-high-pressure metamorphism on the basis of garnet composition and inclusions. Similarly, garnet zones in eclogites have been attributed to prograde to peak growth (Groppo et al. 2009). A special case is represented by garnet in the calcschists (sample C25 and C36), which lack any significant zoning in major and oxygen isotopes. In this case, the poor preservation of garnet (mainly retrogressed to chlorite) may explain the lack of zoning, or the garnet producing reaction in this rock type was a single one.

The presence of metapelitic garnet with inverted and/or abrupt zoning at Lago di Cignana represents a departure from closed-system prograde growth. The rim of garnet in sample C13, C33 and C23 shows such zoning, combined with a low δ^18^O with respect to the prograde growth zones. Reinecke (1998) suggested that the outer zones of garnet in manganiferous schists formed during decompression and greenschist-facies overprint, at temperatures of ~ 400–520 °C. In the tight HP-LT loop experienced by Lago di Cignana (Fig. 6), retrograde garnet growth can occur at the lawsonite out reaction, which is crossed during decompression between 2.0 and 1.5 GPa. Garnet growth at lawsonite breakdown is expected to have high grossular content, as observed for the outermost rim (zone IV, Table 2) in samples C13 and C33, which has an average δ^18^O of ~ 4‰. Inverted and/or abrupt garnet zoning can be explained by open-system behavior and ingress of external fluids, potentially changing the activity of key components (e.g., Mg, Ca) such that the equilibrium garnet mode increases and, in most cases, minor garnet resorption occurs (Giuntoli et al. 2018). A high fluid–flux scenario is required by the large shift in the isotopic composition of inverse zoned garnet in some of the Lago di Cignana samples.

In summary, the process of influx of external fluids that are in chemical and isotopic disequilibrium is considered to be the dominant process in controlling the chemistry of the intermediate to outer part of the metasedimentary garnet in several samples (C23, C13 and C33). It can also be concluded that this pervasive fluid–rock interaction occurred at different stages from peak conditions (i.e. after growth of the garnet core and mantle) to about 1.5 GPa, and was captured by the intermediate and final stages of garnet growth (see details below).

### δ^18^O constraints on fluid–rock interaction

**Fig. 7:**
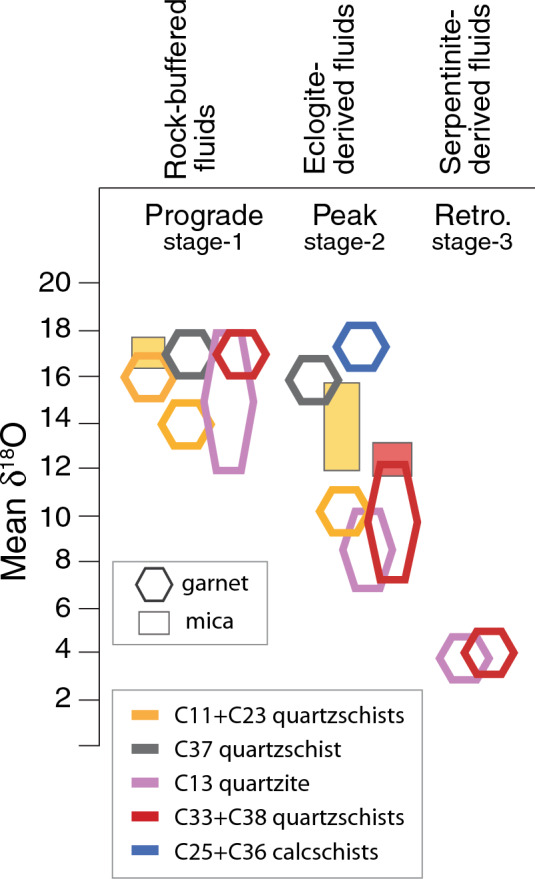
Schematic representation of the mineral isotopic composition at different stages in the metasediments

Garnet in a few samples of metasediments at Lago di Cignana shows a wide range of oxygen isotopic compositions. Within each sample showing isotopic zoning, the general trend is that the δ^18^O of garnet rim is lower than that of the garnet core, with decreases of up to 14‰ (Table 2; Figs. 3, 4). This isotopic variability is also captured by mica in quartzschist C23. An effective mechanism for large shifts in oxygen isotope composition is the infiltration of externally derived fluids with much lower δ^18^O than that of the metasedimentary lithologies. In order to place these fluid infiltration events into the metamorphic evolution, it is essential to relate the in situ oxygen isotope measurements to mineral growth and *P*–*T* conditions. The investigation of sample C33 provides key constraints: (a) inner garnet zones show a prograde growth zoning with increasing pyrope content, which is also observed in sample C37, C23 and parts of C13; (b) the core of large mica grains preserve the highest Si content of 3.55 apfu and probably formed close to the pressure peak, whereas the fine-grained phengite grains defining the foliation and enveloping the garnet have a Si content of 3.4 apfu reflecting lower pressure conditions of 1.5–2.5 GPa (Fig. 6); finally, the texturally later mica with Si < 3.2 apfu formed during exhumation below 1.5 GPa; (c) garnet can be replaced by chlorite upon decompression at pressure bellow 1.1 GPa at greenschist-facies conditions. Combining these observations with the δ^18^O data from garnet and mica of all samples (Fig. 4), the following evolution is proposed (Fig. 7):

*Stage-1*: garnet core (zone I and II) grew during prograde metamorphism in a closed system for oxygen with δ^18^O of 14–18‰; this garnet growth stage is recorded in all metasediments. Given a Δ^18^O_Phe-Grt_ of ~ 2‰ (isotopic fractionation factors from Vho et al. 2019, *T* = 500–600 °C, see details below), it appears that no mica from the prograde stage is preserved, as shown by the generally lower δ^18^O in phengite (Fig. 5), with the possible exception of the highest Si phengite in sample C23, which has δ^18^O of up to 18‰.

*Stage-2*: peak *P* conditions, upon influx of external fluids, garnet with δ^18^O of 7–10‰ formed in equilibrium with high-Si phengite with δ^18^O of ~12‰. This stage is better documented in the micas of samples C23 and C33, where the vast majority of mica has δ^18^O of 12–14‰. This composition is found in mica with progressive decrease in Si and increase in Na content, which corresponds to a decrease in *P* and suggests that mica formed from peak to early stages of decompression. A garnet composition that is in equilibrium with the main mica population is found in zone III of garnet C33 (7–13‰), garnet C13 (7–10‰) and in the rare rims of garnet C23 (zone IV, δ^18^O = 9.9‰). This second observation suggests that, at least for these three samples, mica equilibrated with a fluid at an intermediate stage, where garnet was not growing significantly (volumetrically minor zones) and that during exhumation and deformation to ~ 1.7–1.5 GPa mica recrystallized and adjusted its Si-content without coming into contact with fluids of lower δ^18^O. In other samples where garnet mantle and rim do not record a shift to lower δ^18^O, but maintain the core oxygen isotope composition (calclschist C25 and C36, quartzschist C37, there is no evidence of ingress of external fluid at the peak or retrograde stage.

*Stage-3*: during decompression from 3 GPa and above 1.5 GPa, garnet with δ^18^O ~ 4‰ grew following the influx of fluids with particularly low δ^18^O (see below). This external fluid influx was not recorded by phengite, at least not in the three samples where this mineral was analyzed for oxygen isotopes. The breakdown of lawsonite at ~ 1.5–2.0 GPa is a garnet-forming reaction that we tentatively associate with this final stage of garnet growth, but the ingress of external fluids could have triggered garnet dissolution–reprecipitation at any stage of decompression after stage-2, within the stability field of garnet.

Comparable δ^18^O variability between different samples illustrates the very similar history of the fluid–rock interaction record, although accurate thermodynamic modelling was not possible for most of the samples due to their high Mn content and/or unsuitable assemblage. All metasediments record the prograde stage-1, samples C33, C23 and C13 record fluid influx at the peak stage-2, and the influx of the lowest δ^18^O fluids is recorded in samples C33 and C13 (Fig. 7). An additional fluid circulation event is recorded in the Grt-Czo-Amp-quartzite C13, where the δ^18^O composition of some of the garnet grains increases by up to 1.5‰ from zone I to zone II (Figs. 4, 7), suggesting interaction with high-δ^18^O fluids. Such fluids could easily be derived from adjacent metasediments (especially quartzschists and calcschists), or layers within the same samples during prograde devolatilization reactions (e.g., as previously suggested by Bebout et al. 2013).

### Constraints on fluid sources and fluid fluxes

The likely isotopic composition of potential metasomatic agents—and, hence, potential fluid sources—can be estimated from mineral-fluid equilibrium oxygen isotope fractionation. Using the database of Vho et al. (2019), *T* of metamorphic peak at Lago di Cignana (500–600 °C), and considering the range of compositions of the garnet, garnet-water fractionation (Δ^18^O_Grt-H2O_) is ~− 2.5‰ and the phengite–water fractionation (Δ^18^O_Phe-H2O_) is ~− 0.5‰, with a resulting Δ^18^O_Phe-Grt_ of ~ 2‰. The fluids in equilibrium with the isotopically lightest garnet rims (δ^18^O ~ 4‰) are, thus, predicted to have maximum δ^18^O_H2O_ values of ~ 6.5‰, with lower values are always possible due to incomplete fluid–rock equilibration.

Dehydration of mafic rocks is a viable source of fluid at high pressure (e.g., Vho et al. 2020a) and could provide fluid close to peak conditions. Garnet in the eclogites at Lago di Cignana has δ^18^O values of 7.6– 8.5‰, and at *T* of 500–600 °C the equilibrium fluid would have a δ^18^O of 10.1–10.6‰ and would be closer to the composition of the fluid in equilibrium with the mica in the metasediments. Fluids from mafic eclogites produced near peak conditions at Lago di Cignana may derive from dehydration of glaucophane (2.3 wt % H_2_O), epidote minerals (1.7–2 wt% H_2_O) and/or lawsonite (11.5 wt% H_2_O). Possible lawsonite pseudomorphs were found in the metasediments and in the eclogite boudins within the metasediments (Table 1), but are not observed in the eclogite samples from the larger outcrop at the base of the sequence. Glaucophane is present in the Lago di Cignana eclogites, both as retrogression after omphacite and as isolated crystals in an omphacite-dominated matrix, suggesting that mafic rocks were probably dehydrated immediately prior to exhumation (a reaction similar to Law + Gln = Grt + Omp + Qtz + H_2_O), providing a plausible source of eclogite-derived fluids at high pressure. Thermodynamic modeling of assemblages in the eclogites (Groppo et al. 2009) showed that glaucophane reacted out just before peak conditions were reached and that lawsonite breakdown occurred between 2.5 and 2.0 GPa during early exhumation. While percolation of mafic-derived fluids into the metasediments is a possibility, in the Lago di Cignana unit the amount of mafic material is volumetrically minor compared to the metasediments, and its capacity to generate large amounts of fluids is, therefore, limited. Fluid derived from mafic rocks with intermediate δ^18^O could have been sourced from larger volume of eclogites that are found in the nearby Zermatt–Saas unit, which contain lawsonite and amphibole (e.g., Bearth, 1973; Angiboust et al. 2009; Groppo et al. 2009).

The potential sources of metamorphic fluids with δ^18^O of 6.5‰ or lower are limited, with the most likely candidates being hydrated ultramafic rocks such as the adjacent Zermatt–Saas serpentinites. Antigorite constitutes 80–99 vol% of the mineralogy of the serpentinite samples and has δ^18^O between 1 and 6‰ (Table 3). The in situ serpentine δ^18^O measurements presented here are consistent with previous bulk-rock δ^18^O measurements of serpentinites rocks near Valtournenche (1.2 –3.2‰; Cartwright and Barnicoat 1999). Serpentinites of the Zermatt–Saas unit have experienced brucite dehydration at *T* of 490–540 °C releasing up to 3.4–7.2 wt% water (Kempf et al. 2020). In addition, other serpentinites down slab would have reached antigorite dehydration conditions from ~ 680 °C, releasing 5–12 wt% of water depending on composition (Padrón-Navarta et al. 2013). The range of serpentine δ^18^O values (1–6‰) translates into predicted δ^18^O_H2O_ of ~ 3–8‰ for the water produced during serpentine dehydration (fractionation factors from Vho et al. 2019). Therefore, the lowest δ^18^O fluids released from the surrounding Zermatt–Saas serpentinites or other down slab serpentinites would have the expected composition to drive the dramatic change in δ^18^O at stage-3 measured in the garnet of the Lago di Cignana metasediments (Figs. 4, 7). In particular, the *P*–*T* path of the Lago di Cignana unit intersects the brucite-out reaction in the prograde section, before the *P* peak (Fig. 6), whereas the stage-3 fluid influx occurred during exhumation. It is, therefore, likely that the low δ^18^O fluids were either (i) sourced from the surrounding serpentinites dehydrated at a temperature increase during exhumation (see *P*–*T* path of Bovay et al. 2021a in Fig. 6), or (ii) sourced from the deeper parts of the slab and entered the Lago di Cignana sequence at shallow levels. The conclusion of the ingress of fluids derived from serpentinite supports the study of Halama et al. (2020) and Williams (2019) based on B content and isotopes in mica and tourmaline, respectively. In a sample of Grt-Ph-quartzite, Halama et al. (2020) identified interaction with a high-B, high-δ^11^B, serpentine-derived fluid at or near peak metamorphic conditions. In our study, we can confirm that this interaction is widespread in the Lago di Cignana sequence, but probably occurred at lower pressure conditions, during the later stage of garnet growth upon exhumation (stage-3 above).

Finally, phengite and garnet are in isotopic equilibrium in eclogite sample C31 (Fig. 4): phengite δ^18^O of 10.4 ± 1.1‰ and garnet δ^18^O of 8.5 ± 1.1‰ are consistent with Δ^18^O_Phe-Grt_ of ~ 2‰ at 600 °C, (fractionation factors from Vho et al. 2019). This supports the hypothesis presented above that there is no evidence for interaction with external fluids at high pressure in the eclogite samples, and that the δ^18^O composition of the metamorphic minerals was likely inherited from seafloor alteration of the protolith.

The multi-stage process of water–rock interaction in the LCU samples prevents any reasonable calculation of integrated fluid fluxes, as fluid composition has varied during the evolution of the rocks. The recognized stages of fluid-rock exchange are: (i) minor percolation of sedimentary fluids with δ^18^O > 16‰ is recorded in one metasediment, probably during garnet prograde growth; (ii) fluids from dehydration of mafic rocks (δ^18^O < 13‰) pervasively equilibrated mica and locally garnet at or around the metamorphic peak; (iii) pervasive high-pressure flux of fluids derived from the serpentines (δ^18^O < 6.5‰) from adjacent or lower structural levels within the subducted lithosphere; (iv) and finally interaction with low-B retrograde fluids during exhumation (Halama et al. 2020). Calculation of fluid fluxes cannot be performed in these samples, but isotopic shifts of comparable or smaller magnitude in garnet from metasediments have been reconciled with time-integrated fluxes on the order of 10^5^ cm^3^/cm^2^ (Vho et al. 2020b; Bovay et al. 2021b). Such high fluid fluxes, although pervasive, are close to those reported for metamorphic veins and fractures (Zack and John 2007).

Significant fluid–rock interaction with external fluids would be expected to result in changes in bulk-rock major and trace element composition. Notably, the bulk-rock composition of sample C33 that record the strongest shift in garnet δ^18^O and, thus, high fluid–rock interaction with serpentinite fluid (Supplementary Table 5S) is not significantly altered relative to pelitic/psammitic compositions: this sample has K, Rb, Sr, and Cs contents comparable to the GLOSS composition (Plank 2014). The major element composition of the garnet rim in these two samples converges to X_prp_ ~ 8–15 and large shifts in garnet δ^18^O values do not correspond to significant major element shifts (Table 2). The likely preservation of bulk-rock major element composition suggests metasomatism by high-flux but relatively dilute metamorphic fluids (i.e., fluids with high water content but low ability to modify other elements). Our conclusion contrasts with the suggestion of van Schrojenstein Lantman et al. (2021) that large mass transfers occurred during the formation of the garnet nodules in the quartzitic metasediments of Lago di Cignana. The garnet nodules also preserve a high bulk δ^18^O of ~ 18‰ (Frezzotti et al. 2011), which corresponds to the primary value before high-pressure fluid–rock interaction, found in the metasediments in this study. In addition, sample C37 is adjacent to a garnet nodule of the type studied by van Schrojenstein Latman et al. (2020), but it has a limited record of interaction with external fluids. This information calls into question whether large fluid and mass transfer was actually involved in the formation of the diamond-bearing garnet nodules.

### Localization of fluid flux

Variability in fluid–rock interaction, and thus potentially permeability at the local scale, can be inferred from the different degrees of garnet δ^18^O zoning. Metasediment samples showing evidence of large isotopic shifts are located just above the eclogite–metasediment boundary (C13, C23, and C33 in Figs. 1, 4). Permeability between the impermeable eclogite (see below) and the relatively permeable overlying metasediments can result in flow diversion and flux concentration at boundaries (Ague 2014). The first major oxygen isotope shift (stage-2 above) in this boundary zone is best explained by a fluid that equilibrated with the meta-mafic rocks, either the directly underlying eclogites or portions of mafic units that are not present in outcrop. The second fluid influx event (stage-3 above) requires instead that fluids produced in the even deeper lying serpentinites directly entered the Lago di Cignana metasediments at the eclogite–metasediment boundary, without equilibrating with the underlying eclogite.

On the other hand, garnet in eclogites, calcschists and, to a large extent, Grt-quartzschist C37 does not preserve any evidence for interaction with external fluids, as garnet is not zoned in δ^18^O. For the eclogite and Grt-quartzschist, there is ample evidence that the garnet grew over a range of *P*–*T* stages, as garnet in these samples shows bell-shaped (eclogite) or discontinuous (quartzschist) major element zoning. This observation concurs with what reported for another unit associated with the Zermatt–Saas ophiolite (Theodul Glacier Unit, Bovay et al. 2021b). At this locality, eclogite boudins within metasediments have also remained largely impermeable to the pervasive fluid–rock interaction that affected the surrounding metasediments. Instead, B concentrations and isotopes of retrograde mica from Lago di Cignana eclogites suggest interaction with low-B fluids (i.e., not from serpentinites) during retrogression (Halama et al. 2020), which must have occurred after the garnet growth. At Lago di Cignana, most of the metasediments showing weak or no fluid–rock interaction are located at the top of the sequence, close to the deformed contact with the Fe–Ti gabbro. The presence of a tectonized lithological discontinuity, probably of high permeability, may have channeled fluids at the contact rather than in the nearby rocks.

Despite these anomalies (low fluid–rock interaction at the upper contact and in the basal eclogites), it remains that closely associated metasediments vary significantly in their degree of fluid–rock interaction as recorded by garnet. For example, the two Mn-Grt quartzschists C11 and C23, collected from the same level, have comparable assemblages, but significantly different degrees of fluid–rock interaction. These observations suggest that the permeability of rock types is a transient phenomenon that depends not only on assemblage, but also on more localized processes such as deformation and mineral reactions with negative volume change that produce local permeability (Bovay et al. 2021b).

Finally, it should be noted that there is no correlation between the degree of high-pressure fluid–rock interaction and the degree of hydration during greenschist-facies retrogression. Sample C36 has undergone extensive retrogression, as evidenced by the replacement of garnet by Chl-Qtz aggregates (Fig. 2f), but still preserves in garnet the high δ^18^O of the metasediments. Samples C11 and C33 show large δ^18^O shifts in garnet, but preserve fresh assemblages with negligible garnet retrogression (Fig. 2b, e). This confirms that garnet recorded fluid-rock interaction at HP, whereas fluid infiltration during later exhumation is not recorded by this mineral.

### Potential links to decarbonation

Carbonate-rich metasediments have the potential for high-pressure decarbonation during infiltration of external H_2_O-rich fluids (e.g., Kerrick and Connolly 2001; Connolly 2005; Gorman et al. 2006; Kelemen and Manning 2015; Scambelluri et al. 2004). An open-system, fluid-driven decarbonation model suggests that fluids derived from serpentinite are particularly important in decarbonating oceanic crust (Gorman et al. 2006). This process has been documented throughout the Western Alps, including at Lago di Cignana (Cook-Kollars et al. 2014) where evidence for carbon-rich fluids at high pressure includes microdiamond-bearing fluid inclusions in garnet of the upper parts of the unit (Frezzotti et al. 2011). Extensive carbonate dissolution is a process to explain carbon removal from the subducted slab (Dolejs and Manning 2010). This was documented by a carbon and oxygen isotope study in high-pressure marbles in Greece, where fluids generated in the slab were focused through narrow zones in the marbles (Ague and Nicolescu 2014).

In the Lago di Cignana sequence, the influx of eclogite- and serpentinite-derived fluids, and the large fluid–rock ratios required to produce garnet with δ^18^O of ~ 4‰ have a high potential to generate C–O–H bearing fluids through decarbonation reactions (such as those involved in the formation of microdiamond inclusions; Frezzotti et al. 2011). Reactive permeability due to pore-forming decarbonation reactions can also contribute to the localisation of fluid flow. Notably, the samples that show the most dramatic fluid–rock interaction are Mn-rich metasediments that do not contain any carbonates, but this is not necessarily evidence for decarbonation in the quartz-rich samples. Sample C13 does not show the typical inclusion-rich garnet cores as observed in the calcschists (C 36) and garnet cores have a lower grossular content (10–25% compared to the rims with ~ 35%). This suggests that carbonate was never present in this rock. Of the two Grt-Ph-quartzschists, C38 has no δ^18^O shift in garnet and contains large, idiomorphic carbonate, whereas sample C33 has a large δ^18^O shift in garnet and contains corroded Fe-bearing carbonate. This indicates limited or no carbon dissolution in the Grt-Ph-quartzschists. Thus, the high fluid flux through the contact zone between eclogite and sediments did not lead to any decarbonation of the sediments as observed in a similar setting in Corsica (Piccoli et al. 2018).

Through the detailed study of fluid inclusions in garnet in Mn-rich nodules, Frezzotti et al. (2011) suggested that carbonate dissolution is an important mechanism of carbon release. Garnet in the manganese–garnetite nodules analyzed by Frezzotti et al. (2011) has high δ^18^O of 17.4–17.9‰, which is comparable to that of garnet in quartzschists adjacent to similar nodules located higher in the stratigraphic sequence (sample C37, garnet δ^18^O = 16.1–17.6‰; Table 2). This coincidence suggests minimal interaction of the garnetite with the low δ^18^O fluids documented in our study. Also, across the unit, shifts in garnet δ^18^O are not generally associated with marked increases in garnet Grs/And content, as would be expected if garnet was growing during a reaction involving destabilization of calcium-bearing carbonates. This agrees with an extensive study of calcschists in the Western Alps, from low-grade conditions up to the highest metamorphic grade represented by the Cignana samples, where very limited changes in the carbon inventory were observed (Cook-Collars et al. 2014). While the eclogite- and serpentinite-derived fluids provide a plausible mechanism for decarbonation, a direct link cannot be implied here; further targeted detailed microanalysis may provide such information (including identification of microdiamonds in garnet nodules from this sample set).

## Conclusions

A microanalytical study of the geochemical zoning of garnet and mica within a section of deeply subducted upper oceanic crust at Lago di Cignana has revealed multiple episodes of localized infiltration of external fluids during high-pressure metamorphism. Dehydration of eclogites involving lawsonite and amphibole, and dehydration of serpentinites are identified as the main fluid sources at different *P*–*T* stages. Closely associated metasediments vary considerably in their degree of fluid–rock interaction, suggesting local variations in permeability. Fluid flow was channeled in the lower part of the unit, probably due to the permeability contrast between metasediments and eclogites, but was not detected in the upper part of the unit near to the tectonized contact with metagabbro. The large isotopic shifts in major rock-forming minerals imply large fluid fluxes that occurred at high pressure, relatively close to the metamorphic peak at about 90 km depth, and during high-pressure exhumation. Furthermore, the influx of externally derived aqueous fluids into the metasediments is not directly related to decarbonation reactions.

### Supplementary Information

Below is the link to the electronic supplementary material.

Supplementary file1 (PDF 14305 KB)

Supplementary file2 (XLSX 144 KB)

## Data Availability

All data presented in this paper are given in the Supplementary Tables.
