# Supplementary material for: Tracing fluid infiltration into oceanic crust up to ultra-high-pressure conditions
Source: Contrib Mineral Petrol. 2023 Oct 19;178(11):79. doi: 10.1007/s00410-023-02060-6 (PMC11008078; doi:10.1007/s00410-023-02060-6)
Supplement: Supplementary file 1 — Supplementary file1 (PDF 14305 KB) [file 410_2023_2060_MOESM1_ESM.pdf]

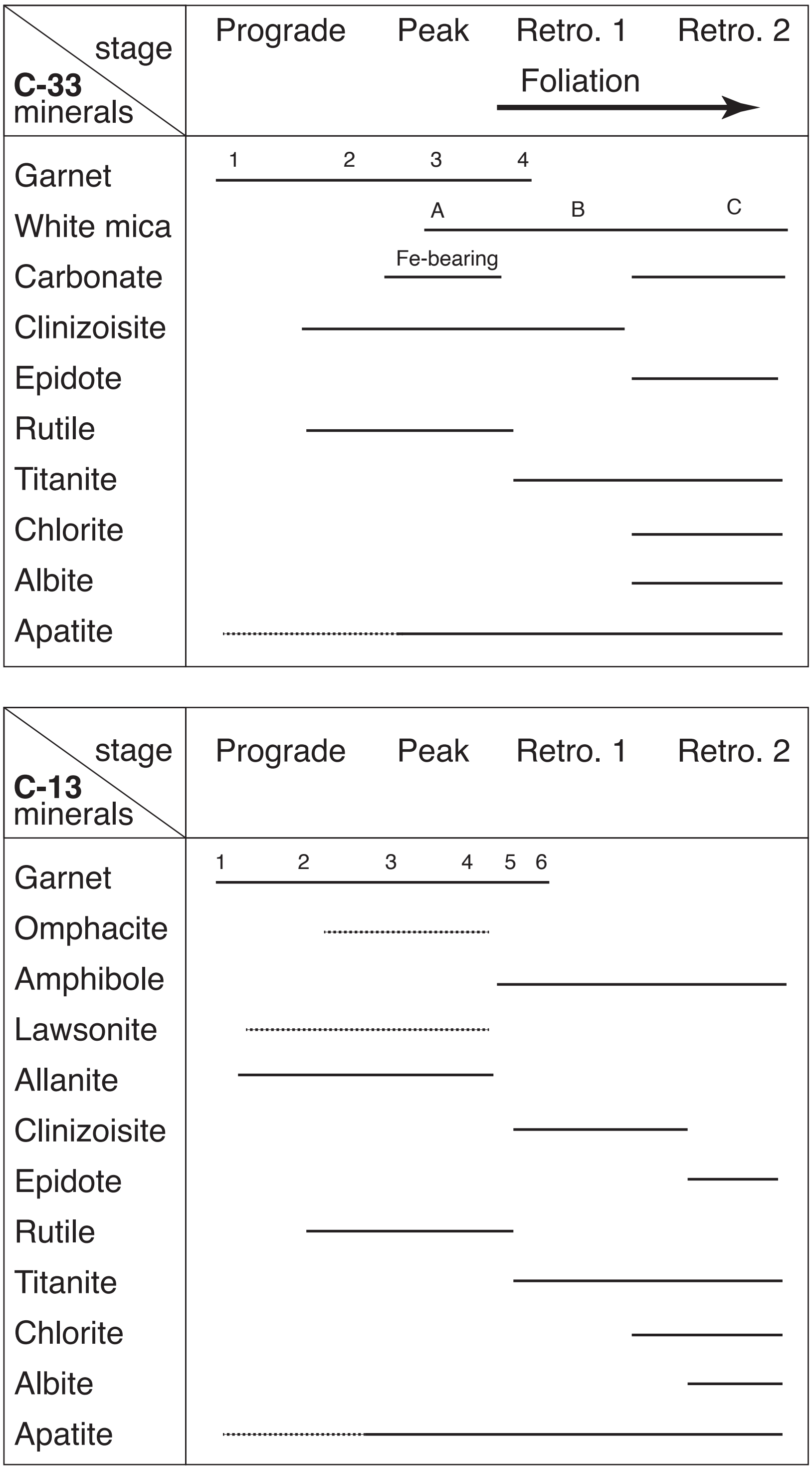

Supplementary Figure 1S. Crystallisation diagrams of minerals with different metamorphic stages for sample C33 and C13.

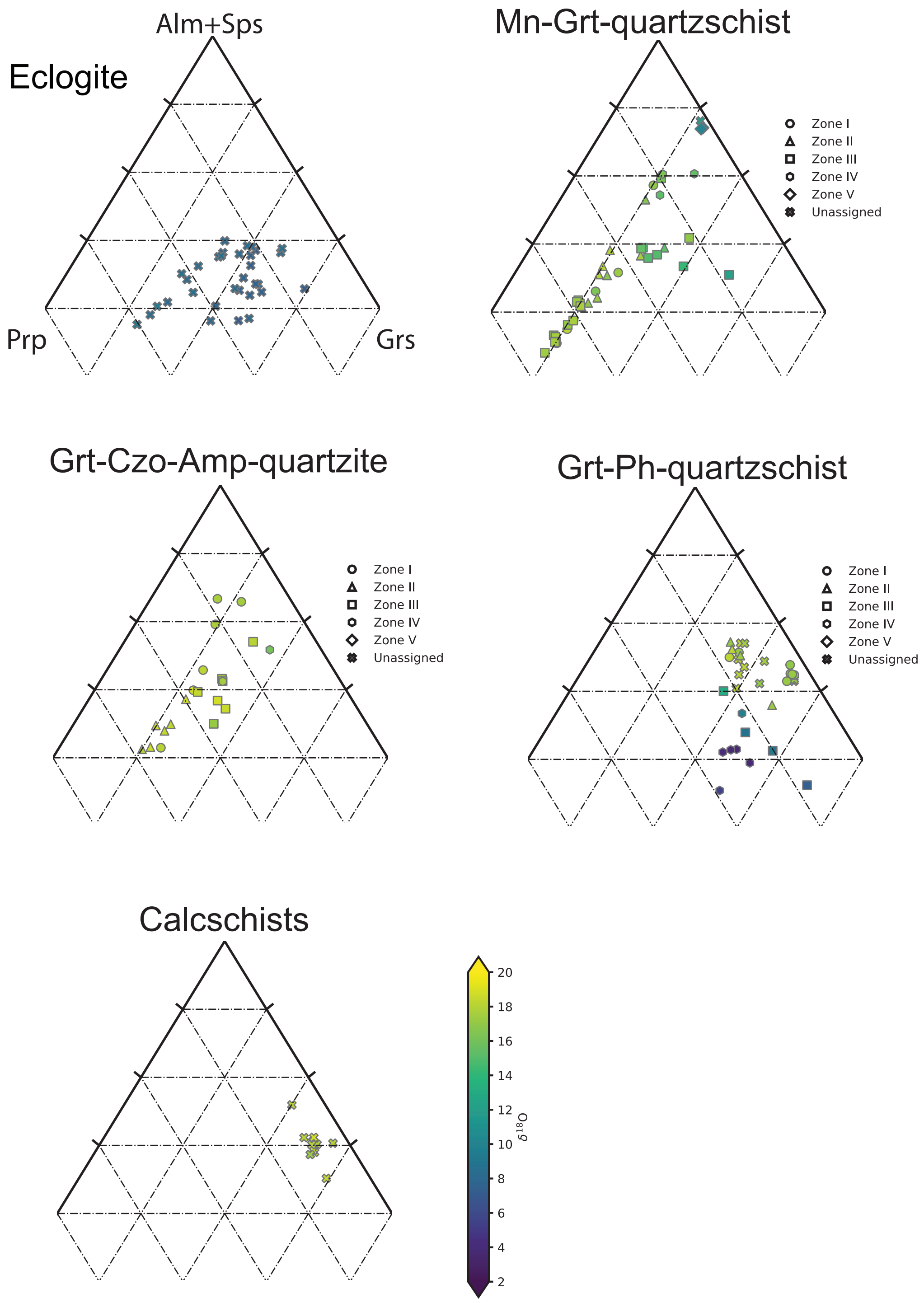

Supplementary Figure 2S. Garnet composition in the different samples plotted on triangular diagrams Alm+Sps – Prp – Grs. Zones are rendered with different symbols.

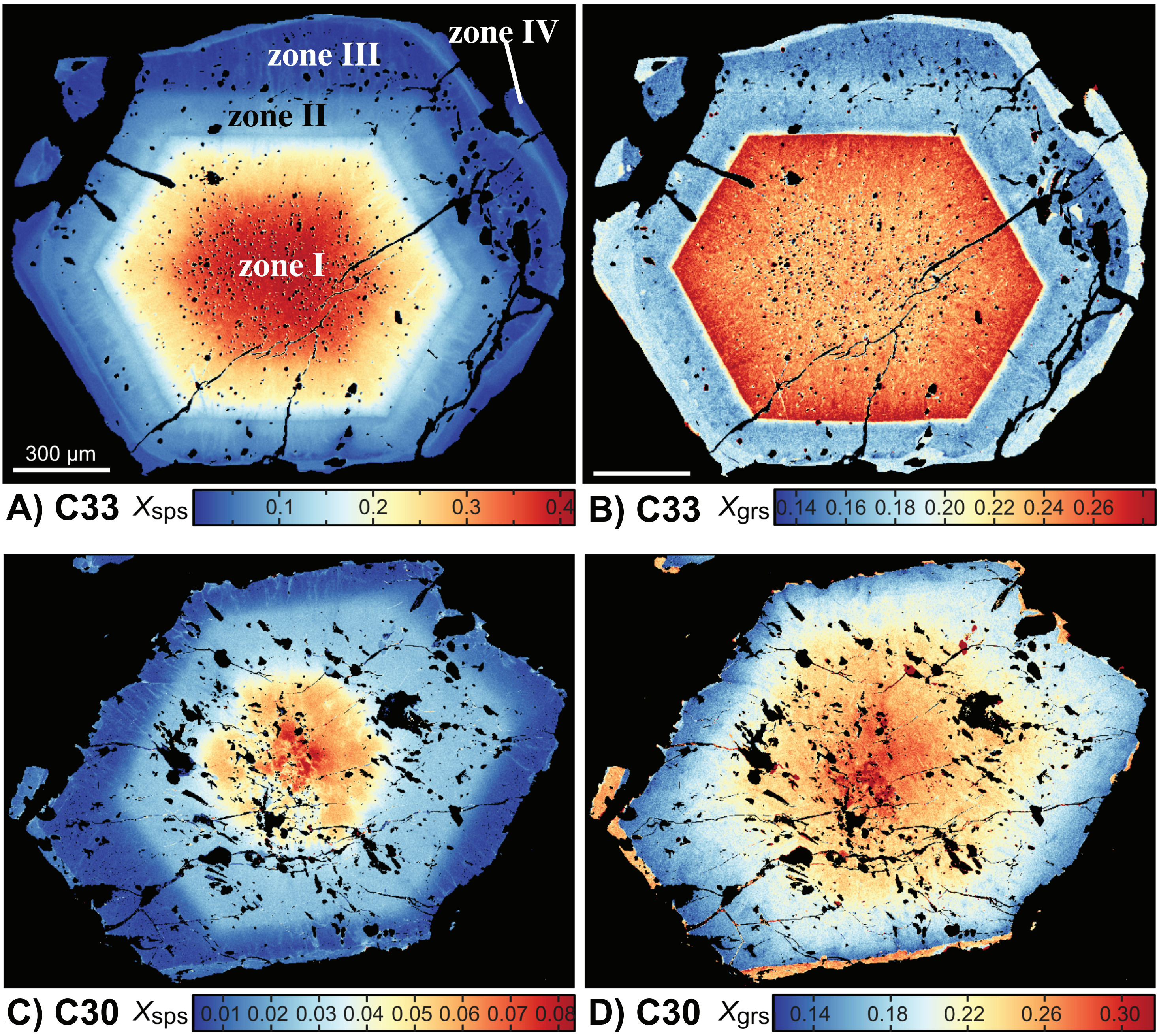

Supplementary Figure 3S. Ca and Mn maps of garnet crystals form metasediment C33 (A, B) and eclogite C30 (C, D). Composition is expressed in Xgrs and Xsps and calculated with the program XMap-Tools (Lanari et al. 2014).

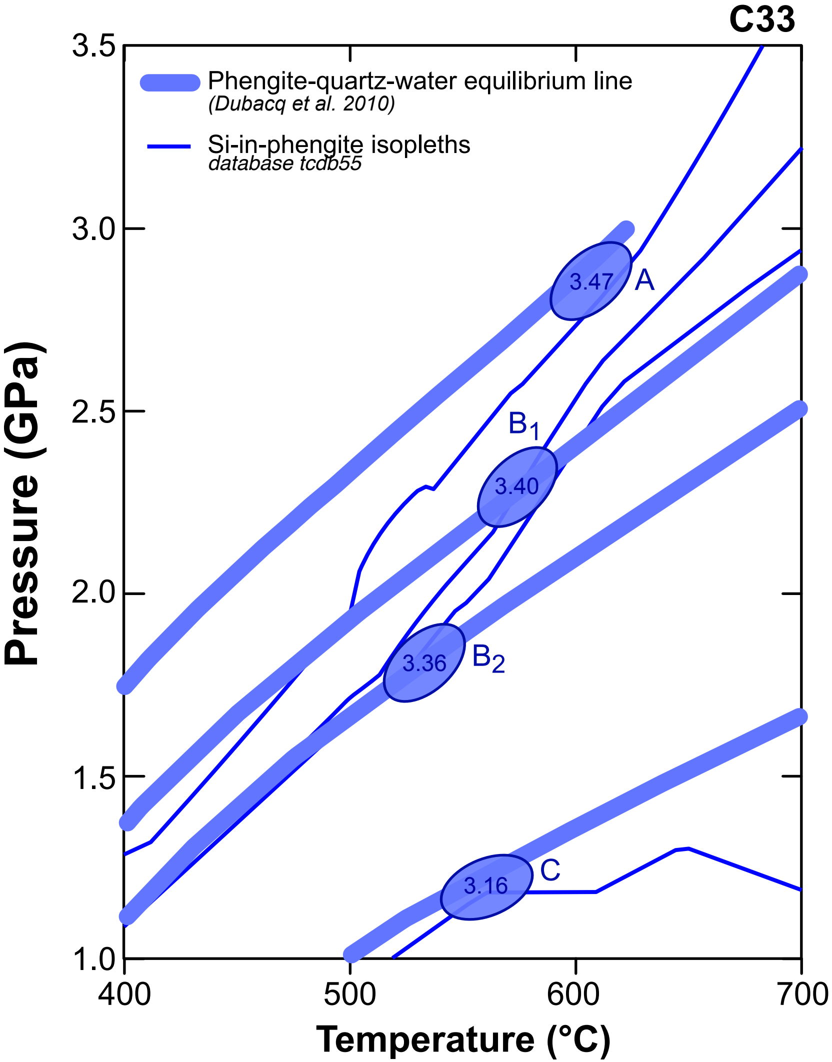

Supplementary Figure 4S. Thermodynamic modelling results for sample C33 showing the Si-isopleths and the P-T lines representing the phengite-quartz-water equilibria used to draw the blue ellipses shown in Figure 6.
